# Consistency and clarity of pharmacogenomic guidance in UK medicine patient information leaflets: A cross‐sectional analysis

**DOI:** 10.1002/bcp.70521

**Published:** 2026-03-15

**Authors:** Parth Narlawar, Chloe Ratcliffe, Videha Sharma, William G. Newman, Munir Pirmohamed, John McDermott

**Affiliations:** ^1^ Manchester Centre for Genomic Medicine St Mary's Hospital, Manchester University Hospitals NHS Foundation Trust Manchester UK; ^2^ Division of Evolution, Infection and Genomics, School of Biological Sciences The University of Manchester Manchester UK; ^3^ Department of Pharmacology and Therapeutics, Wolfson Centre for Personalised Medicine University of Liverpool Liverpool UK; ^4^ Liverpool University Hospital Foundation NHS Trust Liverpool UK

**Keywords:** drug labelling, patient education, patient education handout, pharmacogenetics, practice guidelines

## Abstract

Pharmacogenomic (PGx) information has the potential to support the safe and effective use of medicines, yet there is uncertainty about how this information can be best communicated to patients. Summaries of product characteristics (SmPCs) and patient information leaflets (PILs) for all UK‐approved medicines with strong evidence supporting a PGx prescribing recommendation (CPIC Levels A or provisional A/B) were reviewed, using keyword searches to identify relevant content and estimating the reading age of PGx information in PILs using the Flesch–Kincaid grade level. Among 353 products, 281 (80%) SmPCs contained PGx information, but this was reflected in only 115 (33%) PILs. Where present, the PGx content in PILs required an average reading age of 19.1 years, far exceeding the typical UK reading age of 9–11 years. As the availability of genomic data becomes increasingly routine, the variable PGx information provided in PILs risks undermining patient understanding, engagement and safe personalized prescribing.

**What is already known about this subject**

- Pharmacogenomic (PGx) information is increasingly embedded in regulatory drug documents such as SmPCs internationally, yet prior studies have shown that PGx content is inconsistently translated into patient‐facing materials.
- There is an increasing commitment across health systems to make genomic data available in routine healthcare, meaning more patients and clinicians will encounter PGx results in real‐world prescribing contexts.
- UK regulatory agencies require patient information leaflets (PILs) to be understandable and aligned with SmPCs, but there is currently no standard for when or how PGx information should be communicated.

**What this study adds**

- There is a substantial gap between PGx information in SmPCs and PILs within the United Kingdom, with marked inconsistency both between and within drug classes.
- When PGx information is included in PILs, the wording lacks standardization and requires a high reading age, exceeding recommended levels for patient‐directed health information.
- With genomic data expected to reach more patients through routine care, this work highlights the growing need for clear guidance on when and how PGx information should be incorporated into PILs.

## 1 INTRODUCTION

Pharmacogenomics (PGx) is the study of how an individual's genomic variation influences their response to therapeutic treatments.^1^ PGx‐guided prescribing is increasingly becoming part of routine clinical practice in many health systems, and prescribing guidelines now exist for a large number of commonly used medicines.^2^ In the United Kingdom, there is a significant commitment to personalized medicine, with the NHS 10‐year Health Plan for England aiming to launch a population health service and make results available within the NHS App.^3^

If these ambitions are realized, genomic data will become widespread across healthcare, with both healthcare professionals (HCPs) and patients able to access PGx results.^2^ In this context, clear and trusted information will be required to support the safe clinical use by both HCPs and the public.^4^ This need is heightened by the fact that some medicines with recommended PGx‐guided dosing, such as ibuprofen and codeine, are available over the counter, which limits opportunities for prescribers to tailor treatment directly.

Patient information leaflets (PILs) are legally required for all licenced medicines and explain, in lay terms, how to use a medicine safely. As the primary written resource routinely provided to patients, PILs could serve as a key tool for communicating PGx information. In the United Kingdom, the Medicines and Healthcare products Regulatory Agency (MHRA) requires that PILs be consistent with the summary of product characteristics (SmPC), the formal regulatory document that outlines evidence, indications and safety considerations.^5^ However, SmPC content is not translated directly, meaning clinically relevant PGx information may not always be included in PILs.

This study aims to examine whether PGx information is present in UK‐approved PILs and SmPCs and to evaluate the accessibility of any identified PGx content. Similar studies have explored this topic in Spain and the United States; this study extends this work by providing a UK‐focused analysis and incorporating an assessment of readability.^6^, ^7^ Mapping the extent of PGx information in both documents will help determine where regulatory guidance is being consistently translated into patient‐facing material and where important information may be omitted (Table 1).

## 2 METHODS

### 2.1 Identification of relevant documents

#### 2.1.1 Inclusion and exclusion criteria

Medicines for which there is evidence that genomic data should be used to guide prescribing (CPIC Level A) were included in the document search.^8^ Medicines with a provisional CPIC Level A or A/B recommendation were also included. Additional branded medicines for which no CPIC level had been assigned, but PGx testing is mandated by the MHRA, were also retrieved (Table S1). For each drug (the active ingredient of the medicine), one formulation was selected per company. Where companies produce more than one formulation of the same active substance, the product most recently added was included, and the remainder were excluded. This was done to prevent duplication of data and distortion of analysis. From this subsequent list of medicines, the PILs and SmPCs were extracted.

#### 2.1.2 Search strategy

The UK electronic Medicines Compendium (EMC, Datapharm) search functionality was used to access product documents for all medicines of interest. Documents were exported as PDFs from the EMC and stored offline prior to data extraction. This approach would capture those medicines that were licensed and available in the United Kingdom.

#### 2.1.3 Data extraction

To determine whether each PIL contained PGx information, a keyword search was performed on all extracted documents using the following terms: ‘genetic’, ‘genomic’, ‘gene’, ‘mutation’, ‘mutant’, ‘variant’ and ‘variation’, as well as the specific gene or protein of interest associated with that medicine as defined by CPIC (e.g., CYP2C19 for clopidogrel). These search terms were developed following a preliminary manual review of 20% of the documents, confirming that this vocabulary captured all relevant content.

Documents containing any of these terms were manually reviewed by two independent authors (PN and CR) to determine whether PGx information was genuinely present, as the same terminology frequently appeared in unrelated contexts (e.g., hereditary fructose intolerance). In uncertain cases, reviewers were asked whether a patient with PGx test results would reasonably interpret the information as PGx in nature. References to tests guiding prescribing that were nonspecific for genetics were recorded separately but were not classified as PGx information. Conflicts were resolved by a third reviewer (JM).

### 2.2 Establishing consistency and clarity

#### 2.2.1 Readability analysis

To assess accessibility, the NHS medical document readability tool was used to determine the reading age of the warning section of the PIL and the specific section containing PGx information.^9^ This tool uses the Flesch–Kincaid system, which can be used to estimate a UK reading age.^10^

#### 2.2.2 Consistency of language

The language used within the PILs to convey PGx information was examined through a manual, structured qualitative analysis.^11^ First, all sections of the PILs containing PGx information were identified and extracted. Second, these phrases were reviewed, and recurrent patterns in wording were coded inductively by two authors independently. For example, terminology related to ‘enzyme variation’ *vs*. ‘protein deficiency’ was distinguished. These codes were then organized into broader themes, under which individual products were subsequently grouped.

## 3 RESULTS

### 3.1 The literature pool

A total of 107 gene–drug pairs were identified, comprising 88 unique medicines that met the inclusion criteria based on their CPIC level of evidence. Several medicines were associated with more than one PGx gene (i.e., fluvastatin with *CYP2C9* and *SLCO1B1*), which was assessed as a single entry to avoid duplication (Table S1). PILs were available on the EMC for 68 medicines and 353 products from 104 companies. The 20 medicines for which information was not available were not available for routine use in the NHS (Figure S1). Therefore, 353 PILs and 353 SmPCs were included. Medicines were grouped into six classes (Table S2).

### 3.2 Presence of PGx information

**FIGURE 1:**
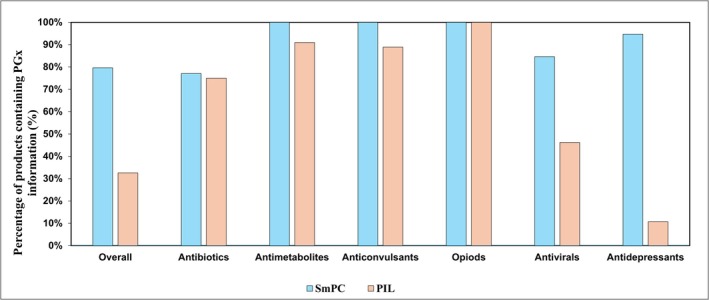
Comparison between PGx information availability in PILs *vs.* SmPCs in major drug classes. From the total 348 PILs and SmPCs, 276 (79%) SmPCs and 113 (32%) PILs contained pharmacogenomic (PGx) information.

Relevant PGx information was present in 281 (80%) SmPCs but only 115 (33%) PILs (Figure 1 and Table S3). Significant inconsistencies were observed both between and within drug classes (Table 1). Only 16 (25%) of medicines had all products include PGx content. Among the 68 medicines, 51 (75%) had at least one SmPC with PGx information, and only 24 of those (47%) had at least one corresponding PIL (Table S3). Of these 24 medicines, four demonstrated intra‐drug inconsistency in PGx information, whereas some product‐specific PILs included PGx content and others for the same active substance did not, indicating a lack of consensus on when inclusion is appropriate. Antidepressants showed the largest discrepancy regarding the inclusion of PGx information (95% of SmPCs *vs*. 11% of PILs).

**TABLE 1:** Drugs identified for analysis where all, some or none of the products contain PGx information in their PILs.

All the products contain PGx information in their PILs (*n* = 20)	Some of the products contain PGx information in their PILs (*n* = 4)	None of the products contain PGx information in their PILs (*n* = 44)	
Abacavir Amikacin Capecitabine Carbamazepine Codeine Dapsone Eliglustat Fluorouracil Fosphenytoin Gentamicin Ivacaftor Mercaptopurine Nitrofurantoin Phenytoin Tetrabenazine Thioguanine Tobramycin Tramadol Valproic acid	Azathioprine Escitalopram Irinotecan Neomycin	Allopurinol Amitriptyline Atazanavir Atomoxetine Atorvastatin Celecoxib Citalopram Clopidogrel Desflurane Divalproex Efavirenz Flurbiprofen Fluvastatin Hydralazine Ibuprofen Isoflurane Lansoprazole Mavacamten Methoxyflurane Meloxicam Netilmicin Nortriptyline Omeprazole	Ondansetron Oxcarbazepine Pantoprazole Paroxetine Peginterferon‐alfa‐2a Pimozide Piroxicam Pitolisant Pravastatin Rasburicase Rosuvastatin Sertraline Sevoflurane Simvastatin Siponimod Suxamethonium Tacrolimus Tamoxifen Velaglucerase alfa Voriconazole Vortioxetine Warfarin

Five medicines mandate PGx testing as part of their marketing authorization: abacavir (HLA‐B57:01*), siponimod (CYP2C9), eliglustat (CYP2D6), mavacamten (CYP2C19) and carbamazepine (HLA‐B15:02*) in specific ancestries. Even here, variation was observed. Abacavir and eliglustat explicitly mentioned the relevant gene, carbamazepine referred to genetic testing without detailing the relevant gene, whereas siponimod and mavacamten referred only to general testing requirements.

### 3.3 Clarity of language

Across the grouped data, 15 distinct phrase types were identified as descriptors of PGx information. The most frequently observed terminology in PILs was ‘Enzyme variation’ (Table S4), which appeared in 28 PILs (25%), followed by ‘Protein/Enzyme deficiency’ (18%) and ‘Mitochondrial mutation’ (16%), highlighting substantial heterogeneity in language. In total, 55 PILs (16%) referred directly to the relevant gene or protein, most often using ‘Gene/protein deficiency’ (Table S4).

**FIGURE 2:**
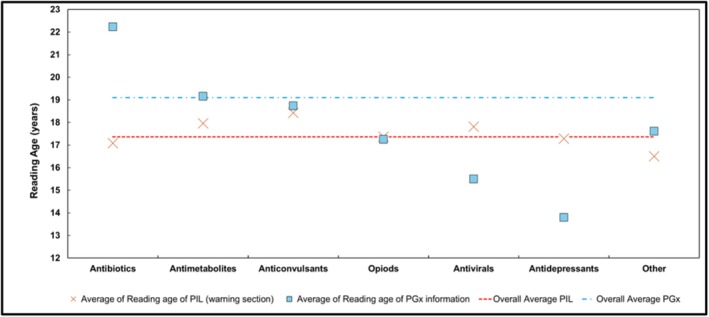
Average reading age of PILs and SmPCs by drug class. Major drug classes were included on the *x* axis, with the ‘Other’ category including glucosylceramide synthase inhibitors, CFTR potentiators, VMAT2 inhibitors and topoisomerase I inhibitors. The average reading age across all PIL warning sections was 17.36 years (red line) compared with 17.36 years for the PGx content in the PILs specifically (blue line).

Readability analysis demonstrated that the mean reading age of PGx information (19.1 years) was considerably higher than both the UK average (9–11 years) and the PIL warning section (17.4 years) (Figure 2). Variation in reading age was greater for PGx sections than for PILs overall. With the exception of opioids, where reading age was comparable between sections, PGx information consistently required a higher reading age than the wider PIL across three of the five main therapeutic classes (Figure 2).

## 4 DISCUSSION

PGx‐guided prescribing has strong supporting evidence and continues to drive global investment in personalized medicine.^12^, ^13^, ^14^ Poor response to medicines is a significant source of morbidity and mortality in the United Kingdom, with adverse drug reactions alone costing the health service an estimated £2.2 billion per year. There is evidence that these adverse events could be significantly reduced through the use of PGx‐guided prescribing, and several population genomics programmes have been established internationally to deliver on this potential.^12^, ^15^ In addition to scaling up genomic testing, programmes must ensure results are communicated clearly to patients and clinicians, supported by consistent and accessible sources of interpretive information. For patients, PILs represent an established and trusted resource; however, this study identifies substantial inconsistencies in PGx content between PILs, including within the same drug class.

In the United Kingdom, the MHRA regulates PILs under the Human Medicines Regulations 2012 (HMR), requiring that they reflect the SmPC and include appropriate precautions and special warnings.^16^ At the same time, MHRA guidance mandates the use of simplified, non‐technical language.^5^ This is particularly relevant for PGx content, which this analysis shows to be one of the most complex areas of the PIL, despite generally low public awareness of PGx.^4^ There is, therefore, a tension between excluding potentially confusing information and providing material that may be essential for safe and effective use. PILs could support public education about PGx, but this requires greater consistency in language, content and clarity.

These findings show variable interpretation of best practice recommendations for PILs. PGx information was present in 33% of PILs compared with 80% of SmPCs. This variation was not consistently attributable to differences between drug classes; within several classes, some products contained PGx information, whereas others did not, including four products where different manufacturers made opposing decisions. Such inconsistency is less consequential when PGx testing is infrequent and not embedded within routine care. CPIC guidance outlines prescribing recommendations only when PGx data are already available rather than specifying when testing should occur.^17^

Under current conditions, the absence of PGx content in PILs may be interpreted as reasonable if patients are unlikely to undergo testing. However, the wider healthcare context is rapidly changing. The NHS 10‐year Health Plan includes making PGx results available via the NHS App, and population‐scale genomic initiatives such as Our Future Health and All of Us are likely to increase the proportion of individuals who know their genetic results.^3^, ^18^ In this future context, the real‐world utility of PGx information in PILs will be considerably greater than historically.

PGx information translated from SmPCs into PILs should be written using accessible language.^19^ Around 7.1 million UK adults read at or below the level of a 9‐year‐old, and NHS guidance recommends that health information should be understandable to an 11‐year‐old.^20^ In this study, PGx sections consistently exceeded this level, and terminology varied widely, including inconsistent reference to the specific gene involved. In future, explicit mention of CYP2D6 would be more useful to a consumer than a vague reference to ‘enzyme variation’. Clear direction is needed regarding both the circumstances in which PGx information should be included and how it should be presented.

These findings align with previous studies from the United States and Spain, which show that PGx information is increasingly embedded within regulatory drug labels but is not communicated to patients in a consistent or standardized way.^6^, ^7^ The convergence of evidence across these healthcare systems highlights a shared need to define clear criteria for when PGx content should appear in patient‐facing documents and how it should be phrased to support understanding and safe use of medicines.

## 5 LIMITATIONS

This study was restricted to products listed in the electronic Medicines Compendium, so a small number of unlisted licenced products may have been missed. It was cross‐sectional and UK‐focused, meaning updates following data collection were not captured. A keyword‐based search may have overlooked PGx content described using atypical terminology, though manual review reduced this risk. Readability statistics are only an approximation of real‐world comprehension, and the study did not assess the practical implications for patient understanding or behaviour.

## 6 CONCLUSION

There is a substantial gap between PGx guidance in UK‐approved SmPCs and the patient‐facing information present in PILs. Where PGx content was included, the language was often inconsistent and written at a reading level beyond that of much of the general population. As PGx results become increasingly available within NHS care and via direct‐to‐consumer testing, the absence of clear and accessible PGx information in PILs presents a risk to safe and optimal use of medicines. Not all PGx recommendations will warrant inclusion in PILs, but where evidence is sufficiently robust, regulatory expectations should support the inclusion of standardized, comprehensible PGx guidance. Doing so would align PILs with the realities of personalized prescribing and support patients and clinicians to make safer, more informed decisions.

## AUTHOR CONTRIBUTIONS

JM and MP developed the concept for the article. PN and CR undertook data collection under the supervision of WGN and JM. Data analysis was undertaken by PN, JM, and VS.

## CONFLICT OF INTEREST STATEMENT

V.S., W.G.N. and J.M. are co‐founders of an early‐stage health technology start‐up, Fava Health Ltd. M.P. currently receives partnership funding, paid to the University of Liverpool, for the MRC Medicines Development Fellowship Scheme (co‐funded by MRC and GSK, AZ, Optum and Hammersmith Medicines Research). He has developed an HLA genotyping panel with MC Diagnostics but does not benefit financially from this. He is part of the IMI Consortium ARDAT (www.ardat.org). No funding from the above declarations contributed to funding for this research.

## Supporting information


**Table S1:** List of drugs with relevant pharmacogenomic gene/drug pairings.
Figure S1: Search strategy flow diagram.
Table S2: Medicines grouped by drug classes.
Table S3: Presence of Pharmacogenomic information in PIL vs SmPC for a given drug.
Table S4: Pharmacogenomic Language used within Patient Information Leaflets.

## Data Availability

The data that support the findings of this study are available from the corresponding author upon reasonable request.
