# Supplementary material for: Consistency and clarity of pharmacogenomic guidance in UK medicine patient information leaflets: A cross‐sectional analysis
Source: Br J Clin Pharmacol. 2026 Mar 15;92(6):1934–9. doi: 10.1002/bcp.70521 (PMC13206309; doi:10.1002/bcp.70521)
Supplement: Supplementary file 1 — Table S1: List of drugs with relevant pharmacogenomic gene/drug pairings. Figure S1: Search strategy flow diagram. Table S2: Medicines grouped by drug classes. Table S3: Presence of Pharmacogenomic information in PIL vs SmPC for a given drug. Table S4: Pharmacogenomic Language used within Patient Information Leaflets. [file BCP-92-1934-s001.docx]

**Supplementary Material for Consistency and Clarity of Pharmacogenomic Guidance in UK Medicine Patient Information Leaflets: A Systematic Review**

**Contents**

1. **Appendix 1:** Supplementary Table 1: List of drugs with relevant pharmacogenomic gene/drug pairings
2. **Appendix 2:** Supplementary Figure 1. Search strategy flow diagram.
3. **Appendix 3:** Supplementary Table 2: Medicines grouped by drug classes
4. **Appendix 4:** Supplementary Table 3: Presence of Pharmacogenomic information in PIL vs SmPC for a given drug.
5. **Appendix 5:** Supplementary Table 4**:** Pharmacogenomic Language used within Patient Information Leaflets.

**Appendix 1**

| **Supplementary Table 1: List of drugs with CPIC Level A Evidence for Pharmacogenomic Guided Prescribing** | | | |
| --- | --- | --- | --- |
| Abacavir  Allopurinol  Amikacin  Amitriptyline  Atazanavir  Atomoxetine  Atorvastatin  Azathioprine  Capecitabine  Carbamazepine  Celecoxib  Citalopram  Clopidogrel  Codeine  Dapsone  Desflurane  Dibekacin^✝^  Divalproex sodium**  Efavirenz  Eliglustat**  Enflurane^✝^  Escitalopram | Fluorouracil  Flurbiprofen  Fluvastatin  Fosphenytoin  Gentamicin  Halothane^✝^  Hydralazine  Ibuprofen  Irinotecan  Isoflurane  Ivacaftor  Kanamycin^✝^  Lansoprazole  Lornoxicam^✝^  Lovastatin^✝^  Mavacamten*  Meloxicam  Mercaptopurine  Methoxyflurane  Methylene blue^✝^  Neomycin  Netilmicin | Nitrofurantoin  Nortriptyline  Oliceridine**^✝^  Omeprazole  Ondansetron  Oxcarbazepine  Pantoprazole  Paromomycin^✝^  Paroxetine  Peginterferon alfa-2a  Peginterferon alfa-2b^✝^  Pegloticase^✝^  Phenytoin  Pimozide**  Piroxicam  Pitavastatin^✝^  Pitolisant**  Plazomicin^✝^  Pravastatin  Primaquine^✝^  Rasburicase  Ribostamycin^✝^ | Rosuvastatin  Sertraline  Sevoflurane  Simvastatin  Siponimod**  Streptomycin^✝^  Succinylcholine  Tacrolimus  Tafenoquine^✝^  Tamoxifen  Tenoxicam^✝^  Tetrabenazine**  Thioguanine  Tobramycin  Toluidine blue^✝^  Tramadol  Tropisetron^✝^  Valproic acid  Velaglucerase alfa**  Voriconazole  Vortioxetine  Warfarin |

**Footnote:** All Medicines where there is evidence that genomic data should (CPIC Level A) be used to guide prescribing have been listed. Additional medicines where there are no CPIC guidelines, but PGx testing is mandated by the MHRA, were also retrieved and are highlighted by an asterisk (*). Medicines without formal guidelines, but where the CPIC level of A or A/B is provisional were also included and are highlighted by a double asterisk (**). Medicines which are unlicenced in the UK or are not available for in NHS clinical practice are indicated by a cross (✝).

**Appendix 2**

**Supplementary Figure 1.** Search strategy flow diagram.


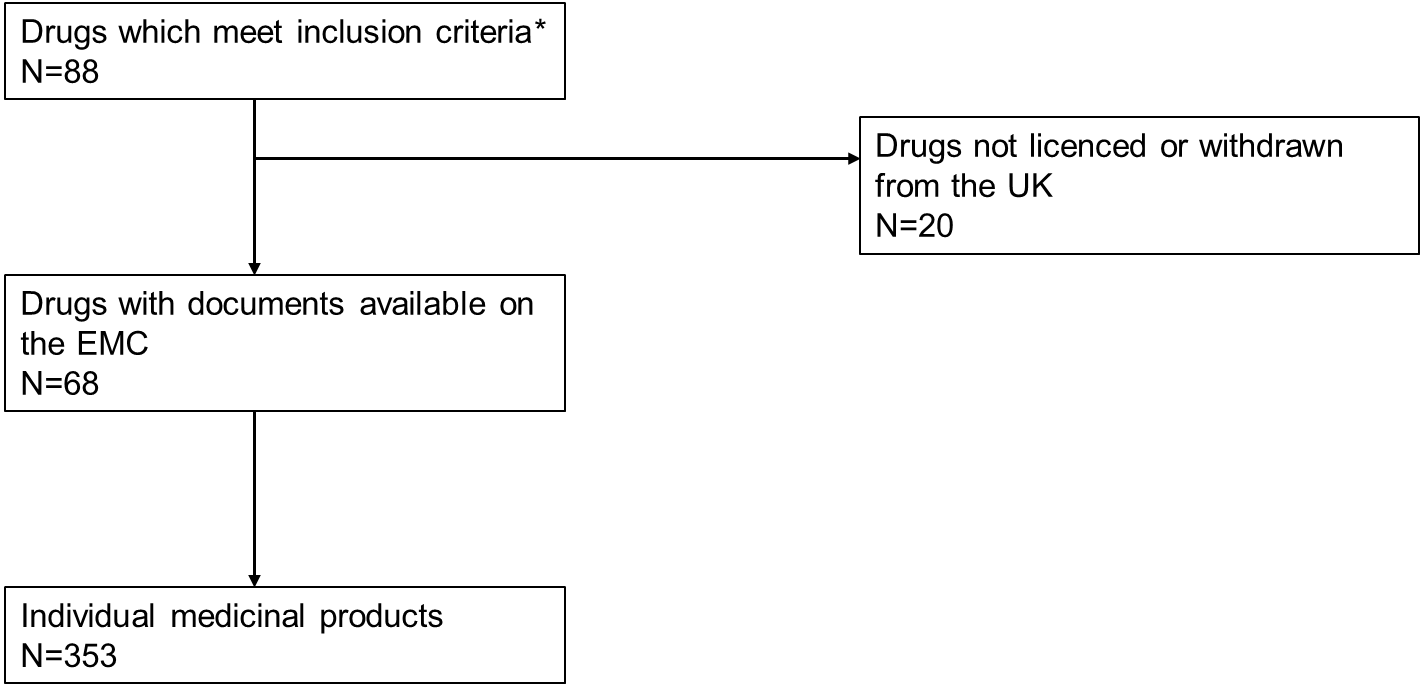


**Footnote:** *Medicines where there is evidence that genomic data should (CPIC Level A) or could (CPIC Level A/B) be used to guide prescribing were included in the document search. Medicines not included in a published guideline, but where there is a provisionally assigned CPIC Level A/B recommendation were also included. Additional branded medicines where there is no assigned CPIC level, but PGx testing is mandated by the MHRA, were also retrieved

**Appendix 3**

**Supplementary Table 2: Medicines grouped by drug classes**

| **Drug classes** | **Drugs** |
| --- | --- |
| Statins | Atorvastatin, fluvastatin, pravastatin, rosuvastatin, simvastatin |
| Antibiotics | Amikacin, gentamicin, neomycin, netilmicin, tobramycin, dapsone, nitrofurantoin |
| Anticonvulsants | Carbamazepine, oxcarbazepine, Fosphenytoin, phenytoin, valproic acid, divalproex sodium** |
| General Anaesthetics | Desflurane, isoflurane, methoxyflurane, sevoflurane, suxamethonium chloride |
| NSAID’s | Flurbiprofen, ibuprofen, celecoxib, piroxicam, meloxicam |
| Antimetabolites | Capecitabine, mercaptopurine, fluorouracil, azathioprine, thioguanine |
| Antidepressants | Vortioxetine, citalopram, escitalopram, paroxetine, sertraline, amitriptyline, nortriptyline, |
| Opioids | Codeine, tramadol |
| Interferons | Peginterferon alfa-2a, peginterferon alfa-2b |
| PPI’s | Lansoprazole, omeprazole, pantoprazole |
| Antivirals | Efavirenz, abacavir, atazanavir |

**Footnote:** Medicines were grouped by drug class where there was >1 medicine in any given class. Branded medicines (Mavacamten, Siponimod, and Eliglustat) were not categorized into classes.

**Appendix 4**

**Supplementary Table 3: Presence of Pharmacogenomic information in PIL vs SmPC for a given drug.**

| **Drug** | **N* of products with PGx information in their SmPC** | **N* of products with PGx information in their PIL** |
| --- | --- | --- |
| Abacavir | 6 | 6 |
| Allopurinol | 9 |  |
| Amikacin | 4 | 4 |
| Amitriptyline | 14 |  |
| Atazanavir |  |  |
| Atomoxetine | 10 |  |
| Atorvastatin | 12 |  |
| Azathioprine | 10 | 8 |
| Capecitabine | 3 | 3 |
| Carbamazepine | 8 | 8 |
| Celecoxib | 4 |  |
| Citalopram | 9 |  |
| Clopidogrel | 7 |  |
| Codeine | 13 | 13 |
| Dapsone | 4 | 4 |
| Desflurane |  |  |
| Divalproex | 1 | 0 |
| Efavirenz | 5 |  |
| Eliglustat | 1 | 1 |
| Escitalopram | 7 | 6 |
| Fluorouracil | 4 | 4 |
| Flurbiprofen |  |  |
| Fluvastatin |  |  |
| Fosphenytoin | 2 | 2 |
| Gentamicin | 9 | 9 |
| Hydralazine |  |  |
| Ibuprofen |  |  |
| Irinotecan | 6 | 4 |
| Isoflurane |  |  |
| Ivacaftor | 1 | 1 |
| Lansoprazole | 5 |  |
| Mavacamten | 1 |  |
| Meloxicam | 1 |  |
| Mercaptopurine | 4 | 4 |
| Methoxyflurane |  |  |
| Neomycin | 2 | 1 |
| Netilmicin |  |  |
| Nitrofurantoin | 10 | 10 |
| Nortriptyline | 10 |  |
| Omeprazole | 8 |  |
| Ondansetron | 11 |  |
| Oxcarbazepine | 3 |  |
| Pantoprazole | 9 |  |
| Paroxetine |  |  |
| Peginterferon alfa-2a |  |  |
| Phenytoin | 10 | 10 |
| Pimozide |  |  |
| Piroxicam | 2 |  |
| Pitolisant | 1 |  |
| Pravastatin |  |  |
| Rasburicase |  |  |
| Rosuvastatin | 22 |  |
| Sertraline | 12 |  |
| Sevoflurane |  |  |
| Simvastatin | 6 |  |
| Siponimod | 1 |  |
| Suxamethonium | 2 | 0 |
| Tacrolimus |  |  |
| Tamoxifen | 5 |  |
| Tetrabenazine | 3 | 3 |
| Thioguanine | 1 | 1 |
| Tobramycin | 8 | 8 |
| Tramadol | 15 | 15 |
| Valproic acid | 3 | 3 |
| Velaglucerase alfa |  |  |
| Voriconazole | 7 |  |
| Vortioxetine | 1 |  |
| Warfarin | 8 |  |

**Appendix 5**

**Supplementary Table 4: Pharmacogenomic Language used within Patient Information Leaflets.**

| Descriptor of PGx information used | n (n=115) | Gene/protein mentioned (n,%) |
| --- | --- | --- |
| “Enzyme variation” | 28 | 2 (7.14%) |
| “Protein/Enzyme deficiency” | 20 | 20 (100%) |
| “Mitochondrial mutation” | 19 | 0 (0%) |
| “Gene Variant” | 8 | 8 (100%) |
| “Gene Presence” | 6 | 6 (100%) |
| “Poor metaboliser” | 6 | 6 (100%) |
| “Metaboliser level” | 4 | 4 (100%) |
| “Mitochondrial disease” | 4 | 0 (0%) |
| “Name of Disease”* | 4 | 0 (0%) |
| “Genetic predisposition to side effects” | 4 | 0 (0%) |
| “Inherited mutation and protein deficiency” | 4 | 4 (100%) |
| “Genetic problem” | 3 | 0 (0%) |
| “Enzyme activity” | 3 | 3 (100%) |
| “Inherited variation” | 1 | 1 (100%) |
| “Gene mutation” | 1 | 1 (100%) |

**Footnote:** Where PGx information was present, common phrase types were identified and PILs were grouped accordingly. Text was also screened for any explicit mention of the gene/protein of pharmacogenomic interest. *All four references were to Stevens Johnson Syndrome related to HLA haplotypes.
